# The influence of enjoyment, boredom, and burnout on EFL achievement: Based on latent moderated structural equation modeling

**DOI:** 10.1371/journal.pone.0310281

**Published:** 2024-09-12

**Authors:** Maojie Zhou, Xuemei Wang

**Affiliations:** School of English Studies, Shanghai International Studies University, Shanghai, China; Golestan University, ISLAMIC REPUBLIC OF IRAN

## Abstract

In recent years, the boom in the field of positive psychology in second language acquisition research has seen an increasing number of scholars focusing on the individual well-being of second language learners alongside their learning effectiveness. Despite this growing interest, there is a need to further investigate the specific emotional factors influencing academic achievement in foreign language learning. This study investigates the impact of three emotions—enjoyment, boredom, and burnout—on academic achievement, and the moderating role of academic buoyancy. Data were collected from 563 college English-as-a-foreign-language (EFL) students from China’s mainland using latent moderated structural equation modeling with Mplus. The results of the latent bivariate correlation analysis showed significant correlations between EFL learning emotions, academic buoyancy, and test performance. In the latent moderated structural equations model, enjoyment and burnout predicted test performance. Moreover, academic buoyancy moderated the relationships between enjoyment and test performance, and between burnout and test performance. EFL test performance was highest when enjoyment and buoyancy were both high, or when burnout and buoyancy were both low. These findings highlight the importance of fostering positive emotions and resilience in language learners to enhance their academic performance, offering valuable insights for educators and policymakers aiming to improve foreign language education.

## Introduction

Historically, emotions in the field of second language acquisition (SLA) were overlooked until the 1980s, when the affective filter hypothesis prompted scholars to acknowledge their role, primarily focusing on anxiety [1, 2]. In recent years, the field of SLA has witnessed a significant development in research on positive psychology (PP), with an increasing number of scholars adopting a whole-person perspective on SL learners, that is, focusing on both their learning effectiveness and individual well-being [2]. And in endorsing the negative aspects, it calls attention to human strengths, virtues, and positive qualities [3]. Accordingly, the SLA emotions observed by researchers are not limited to anxiety but also include enjoyment, shame, and boredom [4, 5], further broadening the range of topics in SLA emotion research.

In China, as a compulsory public course for non-English majors, college English plays an important role in cultivating the foreign language (FL) talents needed for national development, dovetailing with the new requirements for FL proficiency in the national development strategy and the country’s strategy of *Chinese Culture Going Global*. However, for a long time, English teaching at universities in China has been facing the problem of being test-oriented [6, 7]. The undergraduates’ inclination to expedite English test clearance impacts college English instruction. Enhancing language application skills is the course objective, and properly guiding students to treat English test sensibly and take college English courses seriously is the basis of and an effective way to passing the exams.

Emotions have been found to be critical to students’ EFL learning processes and achievement [8, 9]. Recent years have witnessed a burgeoning interest in exploring the combined effects of multiple emotions on EFL learning and academic achievement, such as anxiety, enjoyment, and boredom [10–12], these emotions are limited in comparison to the breadth of emotions students may encounter during EFL learning. In particular, there is unexpected scarce attention to burnout in relation to FL learning [13]. Meanwhile, the available research is scarce and seldom goes beyond looking into relationships between pairs of seemingly contrastive emotions [12]. This underscores the importance of incorporating additional dimensions, such as burnout, to comprehensively investigate their cumulative impact on learning outcomes. Additionally, while previous research has highlighted the role of emotions in EFL learning, it has not adequately addressed how students’ positive attributes, particularly academic buoyancy—an important variable in positive psychology—interact with these emotions to influence FL learning outcomes [14]. Therefore, this study aims to address the aforementioned gap by examining the moderating role of academic buoyancy in the relationship between enjoyment, boredom, burnout, and foreign language academic achievement, thus further enriching the existing literature.

## Theoretical analysis and hypothesis

### Emotions and SLA: A positive psychology perspective

Schools have traditionally focused on students’ cognitive development without considering how emotions regulate learners’ psychological states and affect their academic performance [15]. Although the emotion filter hypothesis has led SLA scholars to recognize the importance of emotions in SL learning, the traditional psychological perspective on SL learners’ emotions has focused too much on negative emotions, such as anxiety. In the early 21st century, as PP has become a worldwide craze in many fields, it has also driven the affective turn in SLA research [1, 16]. Scholars not only distinguish various types of emotions from psychological and physiological perspectives but also highlight the functionality of emotions. Specifically, they tap into the positive traits of individuals to compensate for deficits. Additionally, they explore the positive significance of negative emotions in SL learning, attempting to strike a balance between learners’ strengths and deficits [17].

Most studies on emotions from a PP perspective are based on broaden-and-build theory and control value theory [17, 18]. These theories highlight the polarity of positive and negative emotions and emphasize the important role of positive emotions in well-being and academic performance [2]. Broaden-and-build theory is one of the founding theories of PP and is the theoretical underpinning of SL emotion research. Based on this theory, MacIntyre and Gregersen [19] pointed out that the coexistence of positive and negative emotions is a day-to-day emotional mechanism for language learners. Positive emotions help to develop language learners’ thinking and vision, improve their ability to focus on new things, and motivate them to absorb and construct language resources, whereas negative emotions, such as anxiety, can limit their thought–action resources.

In addition to broaden-and-build theory, control-value theory [18], introduced from the field of educational psychology, has played an important theoretical guiding role in the development of PP in the field of SLA [20–23]. This theory focuses on emotion in an academic context and proposes that academic emotions are awakened by the learning process or learning performance [24], including three dimensions: object focus, valence, and activation: 1) object focus classifies emotions into activity emotions (e.g., boredom and enjoyment) and outcome emotions (e.g., anxiety and sadness); 2) valence categorizes emotions into positive emotions (e.g., enjoyment) and negative emotions (e.g., boredom); and 3) activation groups emotions into high-arousal emotions (e.g., enjoyment and anxiety) and low-arousal emotions (e.g., boredom and depression). The theory also systematically explains the antecedents and consequences of emotions; that is, an individual’s assessment of the controllability and value of academic achievement-related activities or outcomes is the antecedent of academic emotions, which, in turn, has direct and indirect effects on academic activities and achievement.

### Relationships between foreign learning emotions and FL achievement

#### Foreign language enjoyment

In PP, foreign language enjoyment (FLE) is one of the most prevalent positive emotional experiences for learners during FL learning [22]. According to broaden-and-build theory [17], positive emotions, such as FLE, are crucial in the process of SLA. Enjoyment can facilitate the building of resources in language learning with its positive power to broaden individuals’ perspectives and enable them to absorb more in learning language [19]. As defined by Dewaele and MacIntyre [25, 26], FLE is the positive emotion felt by learners after overcoming learning difficulties, completing academic tasks, and achieving psychological needs in the process of foreign language acquisition. FLE, as one of the most typical and common positive emotions experienced by FL learners [4], can hedge against the negative emotions in the FL learning process and is therefore beneficial to facilitate FL learning. Furthermore, according to control-value theory [18], FLE is a positive academic emotion that generates high activation during ongoing learning activities or tasks. It has positive effects on SL learning outcomes, including motivation, engagement, and academic performance in SL learning [8, 27–29].

An empirical study by Dewaele and MacIntyre [26] showed that FLE is negatively associated with anxiety. However, the question of how FLE relates to other FL learning emotions needs to be further explored. In addition, studies have confirmed that FLE is positively related to FL performance. For example, Dewaele and Alfawzan [9] found that FLE positively predicts students’ FL achievement based on a sample of 189 junior high school students in London and 152 Saudi Arabian elementary school students. Using a sample of 1,307 Chinese high school English as foreign language (EFL) students, Li [21] concluded that FLE positively predicts actual EFL achievement and self-perceived EFL achievement. Moreover, compared with EFL anxiety, FLE is more predictive of learners’ long-term development of FL proficiency [30] and is more sensitive to the FL learning environment and, therefore, easier to moderate in teaching [31–33].

#### Foreign language learning boredom

The study of emotions in language learning has burgeoned within in the psychological study of FL learning [32, 34]. However, limited attention has been devoted to FL learning boredom (FLLB) [35–38], despite the confirmation of its prevalence in FL classrooms [36]. Boredom is a “silent” emotion [39], it is easily overlooked by educators and researchers [40–43]. Conducting research on FLLB contributes to an enhanced understanding of negative emotions and enriches theoretical outcomes in EFL learning emotion research. This knowledge is instrumental in exploring effective strategies to establish conducive EFL learning environments, alleviate students’ boredom, optimize their EFL learning status [40, 44], and improve their EFL learning self-regulation ability [45].

As an inhibitory negative emotion, FLLB negatively affects the psychological and behavioral processes of learning [46]. At the psychological level, boredom often co-occurs with a variety of other negative emotions, such as frustration, irritability, and loss of meaning. At the cognitive level, boredom is often accompanied by inattention, reduced learning engagement, and even distorted time perception. At the behavioral level, boredom can seriously hinder the motivation and maintenance of students’ learning [43, 47].

Based on control-value theory, the above negative effects may further lead to lower academic performance [41, 44]. Current empirical studies that verify this causal relationship include only one for college students taking EFL online classes [5] and one for Chinese EFL learners in urban and rural elementary schools [48]. However, the generalizability of this study’s findings still needs further validation; as the differences between online and offline teaching contexts may have different effects on students’ academic emotions and psychological processes of learning (e.g., sense of achievement) [5, 44, 49], the negative predictive effect of boredom on EFL achievement should be verified under different teaching modes. Because of the diverse types and wide distribution of higher education institutions in China, whether the findings of this study are applicable to geographic areas with highly different economic conditions, as well as to different levels and types of higher education institutions, remains to be explored as well.

#### Foreign language learning burnout

Burnout, identified as the prominent negative emotion in FL learning, underscores the necessity for additional empirical studies to further explore its role in FL learning [50]. From a psychological point of view, just like any other profession, students are emotionally and behaviorally engaged in a variety of core activities that can be considered their work. That is, these activities are highly structured and mandatory (e.g., attending classes, completing assignments, and taking exams) and point to a specific goal (e.g., passing an exam) [51]. Hu and Schaufeli [52] emphasized that students need to engage in structured, purposeful, and compulsory learning activities (e.g., attending classes, completing assignments, and taking exams) and are also vulnerable to burnout, which they defined as students’ emotional exhaustion and cynicism toward people or events related to learning, as well as reduced academic efficacy and self-confidence.

Recently, researchers have started to explore EFL learners’ burnout by investigating how it interacts with internal and external factors in EFL learners’ learning language. The role of positive emotions and self-regulatory capacity in mitigating burnout has been explored, emphasizing the importance of fostering resilience and positive emotional experiences in educational settings [53, 54]. In China, English learning is strongly test oriented, so students are more likely to experience burnout, which affects their learning outcomes and well-being. This situation highlights the need for empirical research on EFL learning burnout among students in China [4, 8, 13]. The present study is a response to this call. Given the significance of College English Test Band 4 (CET-4) scores for non-English major college students’ degree certificates and career prospects, the distress experienced in these learning activities may manifest as generalized anxiety, depression, or specific burnout [55]. Thus, choosing EFL burnout as another indicator for EFL academic affect in this study and investigating its relationship with EFL test scores are reasonable.

#### Relationship between academic buoyancy and academic achievement

With the positive turn in SLA research in recent years, researchers have begun to explore the role of learners’ positive qualities in sustaining motivation and interest, guiding students to respond positively to external challenges [56], and fostering and enhancing students’ academic buoyancy. The concept of academic buoyancy was first introduced by Martin and Marsh [57] and refers to the ability of students to successfully overcome the difficulties and challenges they encounter in their day-to-day academic activities. Common adversities encompass temporary episodes of academic underperformance, the stress and pressure associated with learning and testing, low confidence resulting from a poor grade, transient declines in motivation and engagement, and dealing with teachers when receiving poor feedback on a piece of work [58]. Researchers generally agree that academic buoyancy is associated with adaptive responses to adversity, including strengthening positive emotions and weakening negative emotions [59].

Academic buoyancy in SLA is a concretization of the concept of academic buoyancy in EFL learning, which refers to “the ability providing learners with the capacity to negotiate the ups and downs of everyday language learning, sustain prolonged effort, and overcome setbacks on the path to second language (L2) learning success” [60]. After examining the literature thoroughly, we found that the effect of academic buoyancy on academic achievement has drawn the attention of researchers, as the research themes related to academic buoyancy have deepened. Academic performance involves academic achievement, engagement, use of learning strategies, and coping with academic adversity. Academic buoyancy has been shown to optimize academic achievement [61–63]. Academic buoyancy, described as an adaptive response to minor academic adversity, might shield against the detrimental effects of negative emotions on academic achievement. Despite these theoretical propositions, empirical evidence supporting these relationships remains limited [59]. Consequently, our hypothesis posits that academic buoyancy may moderate EFL academic emotions.

Employing Latent Moderated Structural Equation Modeling (LMS), this research comprehensively investigates the correlation between enjoyment, boredom, burnout, and foreign language academic achievement among Chinese EFL learners. LMS is chosen for its efficacy in exploring interactions between latent variables, such as mental health, and academic achievement. This method offers statistical efficiency, requiring estimation of only one parameter, and is accessible through widely-used software like Mplus [64]. By utilizing this approach, the study endeavors to shed light on the complex interplay of EFL academic emotions and the moderating effect of academic buoyancy, contributing to a deeper understanding of factors impacting academic achievement in this context.

**Fig 1:**
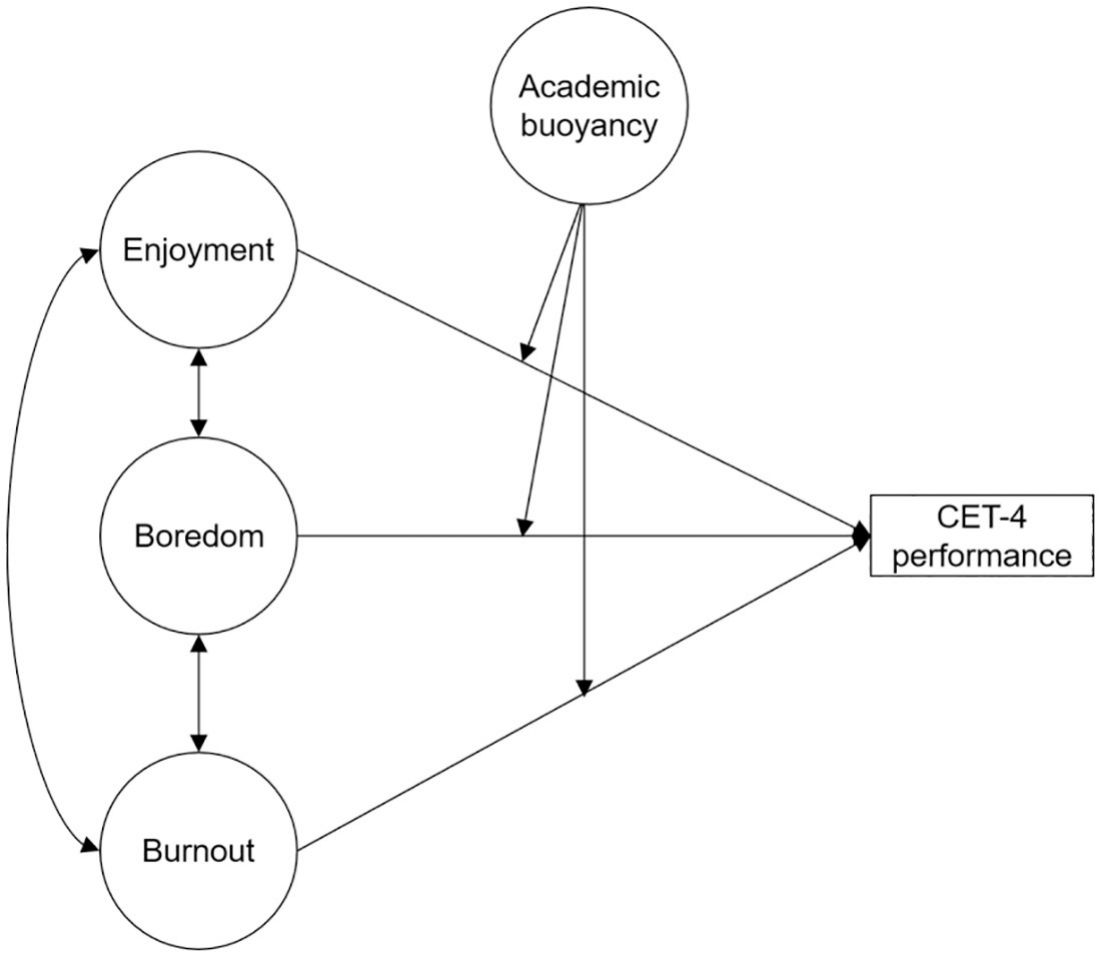
Theoretical model.

Succinctly stated, the present study aims to test the following hypotheses (Fig 1):

*Hypothesis 1*: Enjoyment is significantly negatively correlated with boredom and burnout, whereas boredom is significantly positively correlated with burnout.
*Hypothesis 2*: Enjoyment positively predicts English test performance, whereas boredom and burnout negatively predict English test performance.
*Hypothesis 3*: Academic buoyancy plays a moderating role in the relationship between enjoyment, boredom, and burnout and English language test performance.

## Research methods

### Participants and procedure

This study was formally conducted at the beginning of the second semester of academic year 2021–2022, and the EFL students who participated in this survey were involved in a college English course for at least one semester to ensure the reliability of the research data. Purposive and convenience sampling methods were used, and the questionnaires were distributed to the students in the college English course taught by the first researcher, and then this task was delegated to her colleagues, who were asked to distribute the questionnaires to their college EFL students. Data collection was conducted using both online communication tools and online questionnaire platforms. To ensure a certain degree of privacy for the participants, the researchers informed all respondents that the results would be used for research purposes only. The procedural controls included anonymous responses, confidentiality of responses, and other means, and a common method bias test was conducted on the question items of the scales used through AMOS 24.0, which showed poor model fit and, therefore, a presumption that the data did not have significant common method bias issues.

A total of 589 questionnaires were returned, and after invalid questionnaires (i.e. straightliners) were eliminated, a total of 563 valid questionnaires were retained, with a valid return rate of 95.59%. Among the subjects, 262 (46.54%) were male and 301 (53.46%) were female; 264 (46.89%) were freshmen, and 299 (53.11%) were sophomores. The mean age of the tested students was 19.49 years, with a standard deviation (SD) of 0.589.

### Measures

This study used a quantitative research approach with a combination questionnaire to obtain quantitative data. The instruments are described as follows.

#### Chinese version of the foreign language enjoyment scale

Li et al. [4] localized and revised *The Foreign Language Enjoyment Scale* [25] based on a sample of Chinese high school EFL learners (n = 2,078). The revised scale (Chinese version of the Foreign Language Enjoyment Scale [CFLES]) contains 11 items covering three dimensions: *FLE-Private*, *FLE-Teacher*, and *FLE-Atmosphere*. It is a standard five-point Likert scale (1 = strongly disagree; 2 = disagree; 3 = neither agree nor disagree; 4 = agree; 5 = agree strongly). The scale has high reliability (Cronbach’s α = 0.83) and validity, and it is a reliable and valid localized scale for FLE research in China [4]. In this study, the Cronbach’s α values for the global CFLES and its three subscales were 0.880, 0.750, 0.717, and 0.704, respectively, indicating good reliability. The Kaiser–Meyer–Olkin (KMO) measure of sampling adequacy index was 0.912 (> 0.6), and Bartlett’s test of sphericity was significant (*χ*^*2*^_(*df* = 55, *N* = 563)_ = 4974.181, *p* < 0.001), indicating the appropriateness of the data for factor analysis. CFA showed a good fit to the data, *χ*^*2*^ = 119.793, df = 41, RMSEA = 0.058, CFI = 0.984, TLI = 0.979, SRMR = 0.025.

#### Foreign language learning boredom scale

The Foreign Language Learning Boredom Scale was developed by Li et al. [46] based on the results of a large-scale survey of non-English major EFL students in Chinese universities. A total of 32 questions were included, and each participant responded on a standard five-point Likert scale. The scale differentiates seven main dimensions of FLB: FL classroom boredom, under-challenging task boredom, PowerPoint presentation boredom, homework boredom, teacher-dislike boredom, general learning trait boredom, and over-challenging or meaningless task boredom. In this study, the Cronbach’s α values for the global FLLBS and its seven subscales were 0.972, 0.894, 0.851, 0.800, 0.835, 0.816, 0.855, and 0.788, respectively, indicating high reliability. The KMO measure of sampling adequacy index was 0.949, and Bartlett’s test of sphericity was significant (*χ*^*2*^_(*df* = 496, *N* = 563)_ = 14497.077, *p* < 0.001). The CFA showed a good fit to the data, *χ*^*2*^ = 1200.027, df = 443, RMSEA = 0.055, CFI = 0.947, TLI = 0.941, SRMR = 0.036.

#### Maslach Burnout Inventory-EFL Student Survey

The Maslach Burnout Inventory-EFL Student Survey was finalized by Li et al. [13] based on a sample of Chinese secondary school EFL students (n = 1718) and reformulated the 15-item Maslach Burnout Inventory-Student Survey. It differentiates three main dimensions of EFL students’ burnout: exhaustion, cynicism, and reduced efficacy. A total of 10 questions answered on a standard five-point Likert scale were included. In this study, the Cronbach’s α values for the global Maslach Burnout Inventory-EFL Student Survey and its three subscales were 0.931, 0.851, 0.789, and 0.808, respectively, indicating high reliability. The KMO measure of sampling adequacy index was 0.873, and Bartlett’s test of sphericity was significant (*χ*^*2*^_(*df* = 45, *N* = 563)_ = 3972.914, *p* < 0.001). The CFA showed a good fit to the data, *χ*^*2*^ = 71.227, df = 32, RMSEA = 0.047, CFI = 0.990, TLI = 0.986, SRMR = 0.022.

#### Academic buoyancy scale

This study used *the Academic Buoyancy Scale* [57], which contains four items, to measure the academic buoyancy of non-English major college students. We adapted the four items to the context of the study, for example, “I think I’m good at dealing with pressures (in EFL learning)”. To each item, students rated themselves on a 1 to 5 scale. The KMO measure of sampling adequacy index was 0.857, and Bartlett’s test of sphericity was significant (*χ*^*2*^_(*df* = 6, *N* = 563)_ = 1915.678, *p* < 0.001). The internal consistency reliability of the global ABS was good in this study (Cronbach’s α = 0.838). The CFA showed a good fit to the data, *χ*^*2*^ = 4.151, df = 2, RMSEA = 0.044, CFI = 0.999, TLI = 0.997, SRMR = 0.008.

#### EFL academic achievement

The dependent variable in this study was EFL academic achievement. Specifically, the scores obtained by the EFL students when they took the CET-4 in December 2021 were collected (M = 402.74; SD = 83.624). The CET-4 was chosen as the dependent variable in this study for three reasons. First, the CET-4 is an EFL test with high reliability and validity in China, as it is developed by the Examination Center of the Ministry of Education of China. Second, the theoretical perspective of this study, PP, focuses on the relationship between students’ academic emotions and academic achievement. Compared with general EFL proficiency tests or final exams, the CET-4 is more difficult, has a wider range of knowledge examined, and is more prominently important to most Chinese EFL learners; therefore, it is more possible to trigger negative emotions in learners [65]. Third, the CET-4 is one of the most widely tested English exams in China and has had a significant impact on the teaching of English courses, with some universities linking it to degree certificates and integrating it into the English curriculum. Therefore, the selection of CET-4 scores that match the syllabus and course objectives fits the concept of academic achievement.

### Data analysis

The data from this study were analyzed mainly using SPSS 26.0 and Mplus 8.3. The analysis process was as follows. First, a descriptive statistical analysis of the samples was performed using SPSS to obtain the mean and SD of each dimension.

The factor structure of the scales used was examined using CFA, and bivariate correlations were estimated between all latent variables. Second, structural equation modeling (SEM) was used to estimate the interrelationship between EFL academic emotions and test performance. The bootstrap method was also used to examine the moderating effect of academic buoyancy on the relationship between academic emotions and test performance.

Following the recommendations of Hu and Bentler [66], the following model fit indices were used for evaluation: Chi-square/df (1 < *χ*^*2*^ / df < 3), RMSEA < 0.08, CFI > 0.90, and TLI > 0.90.

## Results

### Descriptive statistics

We examined gender and grade differences in FLE, boredom, burnout, academic buoyancy, and CET-4 performance among EFL college students. The results of the MANOVA tests are shown in Table 1.

**Table 1:** Tests of gender and grade differences in foreign language learning emotions, academic buoyancy, and test performance.

		Enjoyment *M(SD)*	Boredom *M(SD)*	Burnout *M(SD)*	Academic buoyancy *M(SD)*	CET-4 performance *M(SD)*
gender	male	3.801(0.052)	3.669(0.045)	3.805(0.050)	3.171(0.075)	398.66(5.166)
	female	3.763(0.048)	3.718(0.042)	3.845(0.047)	3.151(0.070)	406.299(4.819)
*F*		0.289	0.630	0.343	0.037	1.169
η^2^		0.001	0.001	0.001	0.000	0.002
grade	Freshman year	3.698(0.051)	3.745(0.044)	3.871(0.05)	3.207(0.075)	390.333(5.101)
	Sophomore year	3.853(0.048)	3.651(0.042)	3.787(0.047)	3.119(0.07)	413.702(4.793)
*F*		4.802*	2.392	1.471	0.746	11.147**
η^2^		0.008	0.004	0.003	0.001	0.019

Note.

*p<0.05,

** p <0.01.

There were no significant differences in the scores for enjoyment, boredom, burnout, academic buoyancy, and CET-4 performance among students of different genders. There were also no significant differences in the scores of FLLB, burnout, and academic buoyancy among students in different grades; there were significant differences in the scores for enjoyment (F_(1,547)_ = 4.802, η^2^ = 0.008, *p* < 0.05) and CET-4 performance (F_(1,560)_ = 11.147, η^2^ = 0.019, *p* < 0.01) among students in different grades.

### Latent bivariate correlations

We constructed a measurement model encompassing EFL achievement emotions, academic buoyancy, and performance on the CET-4 test to evaluate latent bivariate correlations. Grade (0 = freshman year, 1 = sophomore year) was added as a covariate and treated as a manifest variable.

The CFA showed a good fit to the data, *χ*^*2*^ = 3125.200, df = 1578, RMSEA = 0.042, CFI = 0.941, TLI = 0.938, SRMR = 0.063, and we then analyzed the correlation coefficients (Table 2). It was found that enjoyment and academic buoyancy showed positive correlated with CET-4 test performance, whereas boredom and burnout were negatively correlated with it. Sophomores reported higher EFL enjoyment and higher CET-4 test performance.

**Table 2:** Correlations between the study variables.

	1	2	3	4	5	6
1. enjoyment	—					
2. boredom	-0.146**	—				
3. burnout	-0.235***	0.860***	—			
4. Academic buoyancy	0.198***	-0.170**	-0.161**	—		
5. CET-4 performance	0.204***	-0.381***	-0.449***	0.242***	—	
6. grade	0.092*	-0.065	-0.051	-0.036	0.140**	—

Note.

*p< 0.05.

**p< 0.01.

***p< 0.001.

### Moderating effect of academic buoyancy on the relationship between FL academic emotions and test performance

We employed the LMS approach implemented in Mplus 8.3 [67] to estimate interactions between academic buoyancy and enjoyment, boredom, as well as anxiety in a single model. First, we tested the baseline model without an interaction term. In this study, the missing values were processed using the method of maximum likelihood, which preserves the existing data as much as possible, resulting in better unbiased parameter estimates and more accurate standard errors (SEs) [68]. SEM (M0) was constructed using enjoyment, boredom, and burnout as the predictor variables, academic buoyancy as a moderating variable, test performance as an outcome variable, and grade as a control variable.

In turn, the model (M1) with the latent moderated term was tested. Absolute model fit indices are not provided in the LMS approach. However, establishing whether a model including an interaction term offers a better fit to the data using relative fit indices [69], such as the Akaike information criterion (AIC) and the Loglikelihood ratio test, is possible.

**Fig 2:**
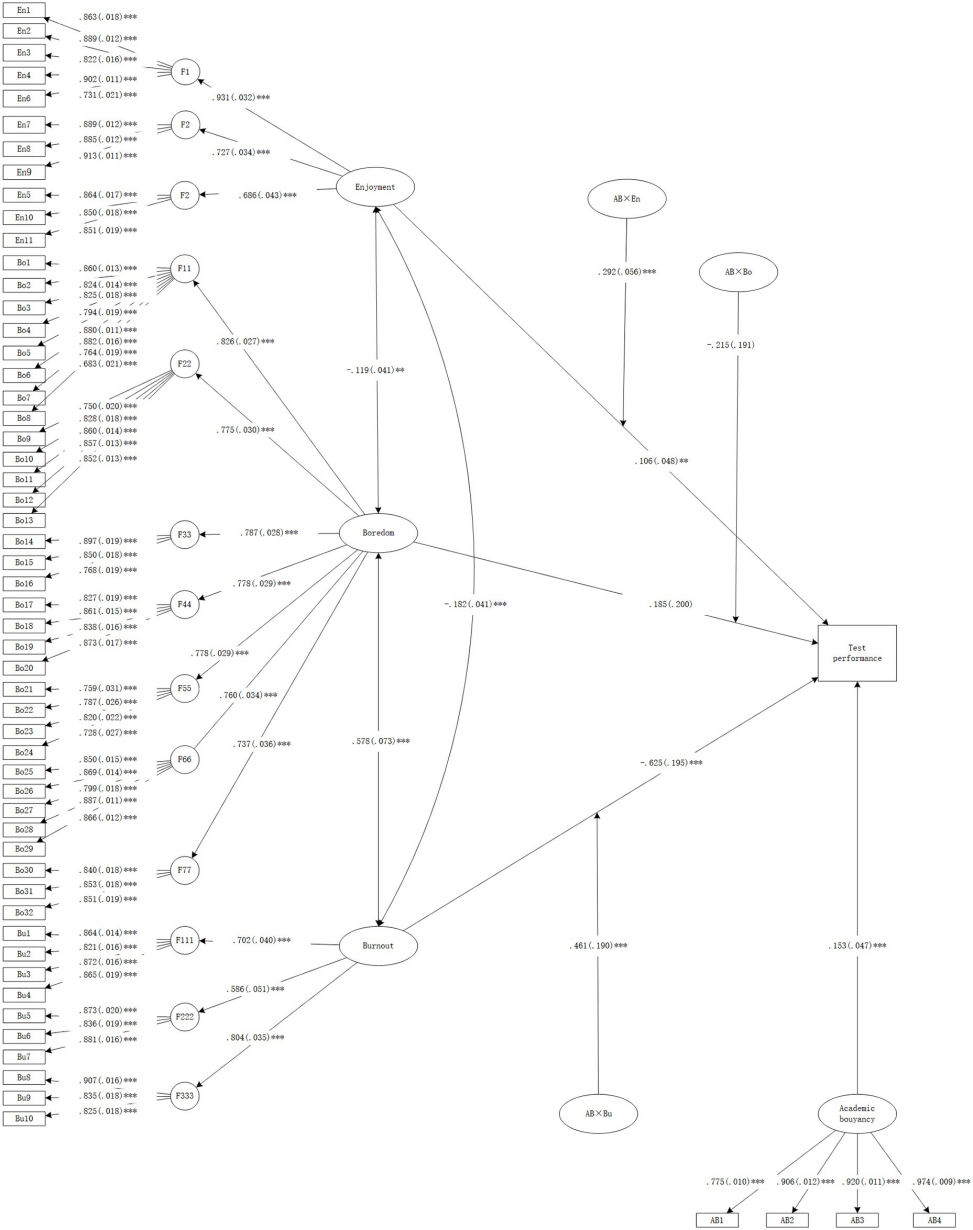
The LMS model. *Note*. Path values are the standardized path coefficients (standard errors); ***p<0.001, **p<0.01, *p<0.05. Grade was controlled.

A better model fit is indicated by smaller AIC and Bayesian information criterion (BIC) values [70] and a larger R-Square (*R*^*2*^) in explaining the variance of the outcome. In this study, the model (M1) including three interaction terms (academic buoyancy × enjoyment, academic buoyancy × boredom, academic buoyancy × burnout) showed an improved fit (ΔAIC = −67.168, ΔBIC = −54.167) and explained a greater proportion of variance in CET-4 test performance (*ΔR*^*2*^ = .0197). Furthermore, a statistically significant likelihood ratio test, *D*(1) = 37, *p <* 0.001, indicated a worse fit for the model (M0) without the three interaction terms. The LMS model is shown in Fig 2.

In the LMS model, there were significant direct predictive relationships between enjoyment and test performance (B = 0.106, SE = 0.048, *p* < 0.01), burnout and test performance (B = −0.625, SE = 0.195, *p* < 0.001), and academic buoyancy and test performance (B = 0.153, SE = 0.047, *p* < 0.001). Of these, enjoyment and academic buoyancy were the positive predictors, whereas burnout was a negative predictor.

The structural coefficients are shown in Table 3. Statistically significant interactions were observed between academic buoyancy and enjoyment, as well as between academic buoyancy and burnout. However, no significant interaction was found between academic buoyancy and boredom.

**Table 3:** Moderating effect of academic buoyancy.

Path Relationship	Academic Buoyancy	Estimate	*S*.*E*	*Est*.*/S*.*E*.	*P*-Value	Lower 2.5%	Upper 2.5%
Enjoyment→CET-4 performance	Low(M-1SD)	-16.351	7.592	-2.154	0.031	-31.231	-1.471
	Medium(M)	9.361	5.240	1.786	0.074	-0.910	19.631
	High(M+1SD)	35.072	7.112	4.931	0.000	21.132	49.012
Boredom→CET-4 performance	Low(M-1SD)	39.870	35.953	1.109	0.267	-30.598	110.337
	Medium(M)	18.443	19.949	0.924	0.355	-20.658	57.543
	High(M+1SD)	-2.984	15.103	-0.198	0.843	-32.586	26.618
Burnout→CET-4 performance	Low(M-1SD)	-116.762	39.867	-2.929	0.003	-194.902	-38.623
	Medium(M)	-67.177	22.058	-3.045	0.002	-110.411	-23.943
	High(M+1SD)	-17.592	16.794	-1.048	0.295	-50.509	15.324

**Fig 3:**
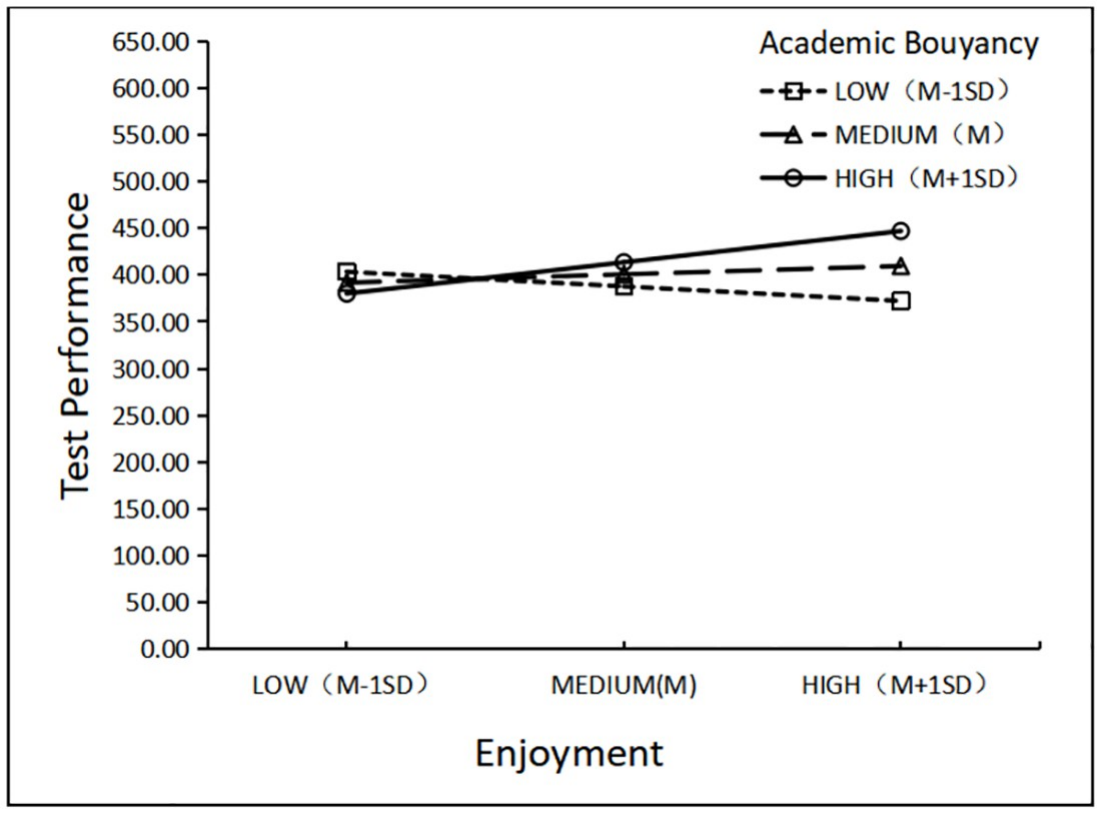
The model-implied effect of academic buoyancy × enjoyment interaction on CET-4 performance.

**Fig 4:**
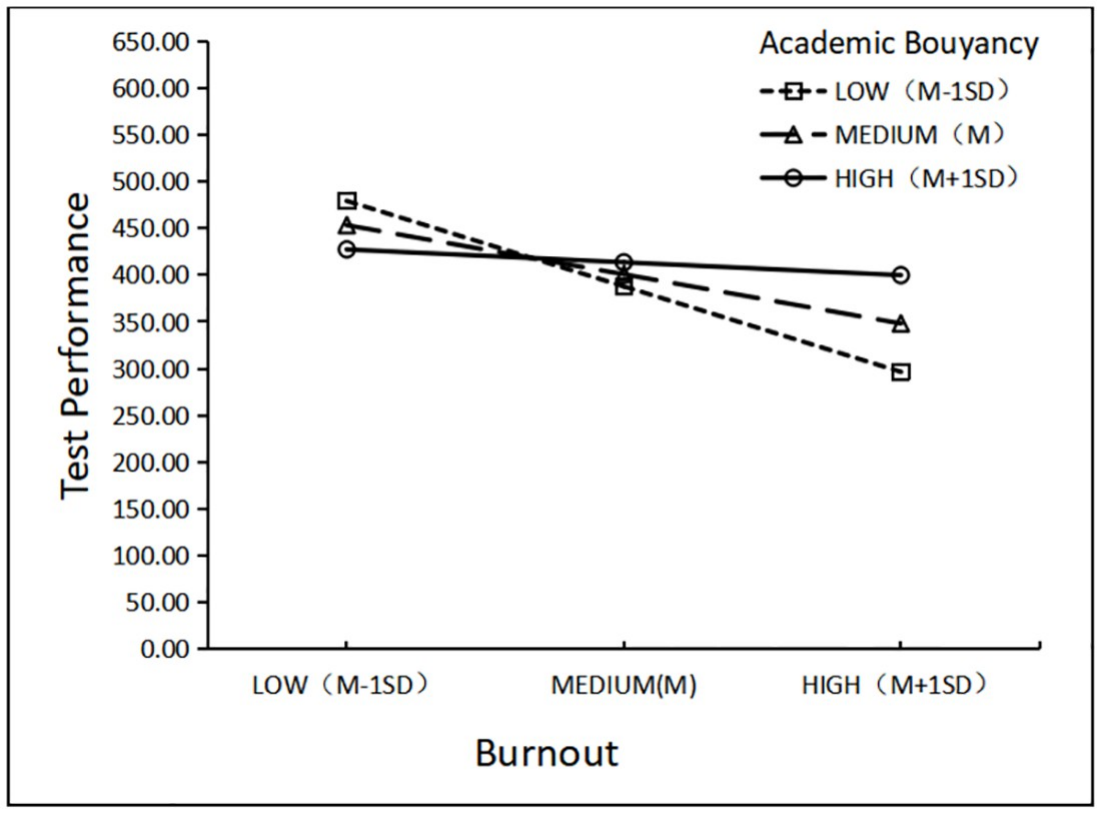
The model-implied effect of academic buoyancy × burnout interaction on CET-4 performance.

To facilitate interpretation of the interaction, we also plotted the relationship between academic buoyancy and test performance at ±1 SD enjoyment (Fig 3) and ±1 SD burnout (Fig 4).

At −1 *SD* academic buoyancy, a negative relationship was observed between enjoyment and test performance (*B* = −16.351, *SE* = 7.592) (Table 3). This relationship was not significant at mean anxiety (*B* = 9.361, *SE* = 5.240) and became positive at +1 SD academic buoyancy (*B* = 35.072, *SE* = 7.112). The finding suggests that academic buoyancy had a negative effect on low EFL enjoyment but a favorable effect on high EFL enjoyment. In other words, in the presence of low EFL enjoyment, high academic buoyancy resulted in lower test scores than low academic buoyancy did. In the case of high EFL enjoyment, those with high academic buoyancy had higher test scores than those with low academic buoyancy. According to the model, increasing EFL enjoyment had a very different effect on EFL students with different levels of academic buoyancy.

At −1 SD academic buoyancy, a negative relationship was observed between burnout and test performance (*B* = −116.762, *SE* = 39.867). This relationship was still negatively significant at mean academic buoyancy (*B* = -67.177, *SE* = 22.058) and became not significant at +1 SD academic buoyancy (*B* = −17.592, *SE* = 16.794). This finding suggests that burnout had almost no effect on higher academic buoyancy but had a greater negative effect on lower academic buoyancy. In other words, in the case of low burnout, high academic buoyancy was associated with lower test scores than low academic buoyancy was. In the case of high burnout, high academic buoyancy was associated with higher test scores than low academic buoyancy was. Based on this model, reducing burnout was evidently better for EFL students with low academic buoyancy.

## Discussion

### Correlation between foreign language enjoyment, boredom, and burnout

The results of the latent bivariate correlation in this study refined the network of relationships between SLA emotions. Our findings affirm the undoing hypothesis of broaden-and-build theory [71, 72], which states that positive emotions *undo* the lingering effects of negative emotions. The theory also posits that positive emotions, such as enjoyment, contribute to the broadening and building of personal resources, fostering resilience and engagement in learning contexts [19]. Thus, enjoyment not only enhances well-being but also serves as a critical buffer against the negative effects of boredom and burnout.

Moreover, we found a positive correlation between FLLB and burnout. In control-value theory, boredom is theorized to impair performance and achievement by undermining interest and intrinsic motivation, reducing cognitive resources, and promoting superficial learning [59]. A possible explanation is that, in a university setting, boredom predicts a lack of engagement in learning activities. Students who lack positive and inactive affects and attitudes regarding their work (e.g., low study engagement) are more vulnerable to burnout than students with high academic engagement [73]. The results of this study add new information to the existing literature about the relationship between EFL learning emotions, such as boredom, and other EFL learning emotions.

### Correlations between FL academic emotions and academic achievement

The findings of this study provide new insights into the question of the predictive role of EFL emotions in academic performance by simultaneously examining enjoyment, boredom, and burnout within a single analytical framework, the research sheds light on their differential effects. It was found that enjoyment and burnout had predictive effects on EFL achievement, no significant association was observed between boredom and EFL performance.

EFL learning enjoyment, defined as the sense of accomplishment and reward derived from overcoming challenges in new and challenging environments, significantly correlates with personal well-being and development [25]. From the perspective of broaden-and-build theory, enjoyment, a positive activating emotion, can broaden students’ thought–action repertoires, thereby enhancing academic achievement though promoting interest and intrinsic motivation, maintaining cognitive resources, focusing attention on the task at hand, and supporting use of flexible and deep learning strategies as well as self-regulation of learning [19, 59]. In addition, control-value theory posits that student learning is influenced by achievement emotions, such as enjoyment and burnout [18]. While acknowledging the existence of a relationship between FLLB and academic performance, as shown in Table 2 and consistent with Li and Han [5] and Li et al. [48], our results emphasize that enjoyment and burnout play a more pivotal role when integrated into the model. Notably, burnout exhibits stronger predictive power than enjoyment. In summary, our findings offer partial support for the second hypothesis. This differential impact underscores the nuanced nature of academic emotions, as described by control-value theory, where the valence and activation level of emotions differentially influence academic outcomes. Essentially, in terms of the three-dimensional taxonomy of control-value theory, enjoyment is a positive activating activity-focused emotion. Higher enjoyment fosters interest, diminishes burnout and boredom, thereby enhancing EFL exam performance [74].

This result partially corroborates the findings of the empirical studies by Li and Han [5] and Li et al. [75]. On the one hand, we validate broaden-and-build theory and control-value theory that positive academic emotions contribute to academic achievement, whereas negative academic emotions undermine academic achievement [18]. In other words, the more pleased the students feel, the less likely they are to feel burned out and bored; as a result, they perform better on exams. The triangular relationship between enjoyment, burnout, and English test performance confirms previous research [8], highlighting both the influence of emotions on academic performance and the association and interaction between positive and negative emotions, as well as providing support for broaden-and-build theory and control-value theory in the EFL learning context. On the other hand, the inclusion of boredom not only expanded the scope of the study but also further validated the applicability of the two theories in the EFL learning context. Importantly, beyond reinforcing previous findings, our study unveils novel insights into the nuanced interplay and distinct contributions of these emotions, advancing our understanding of their intricate relationship with EFL academic achievement.

The differences in the relationships between the three EFL learning emotions and test performance may be attributed to the different intrinsic characteristics of these emotions. According to the three-dimensional taxonomy of academic emotions in control-value theory [24], EFL learning enjoyment is an activity-focused and high-arousal positive emotion, boredom is an activity-focused and low-arousal negative emotion, and burnout is an outcome-focused and low-arousal negative emotion. Currently, the test-oriented phenomenon of EFL teaching in Chinese universities is prominent [7]. Chinese students may also be under the pressure of performing academically perfectly to live up to the expectations of their significant others and to maintain their own social identity [13]. In this teaching environment, the main purpose of students in learning English is to pass exams. Therefore, test scores are more closely related to outcome-focused academic emotions, such as burnout.

### The moderating effect of academic buoyancy

Academic buoyancy has gradually attracted attention in the field of EFL learning and teaching [62]. From a conceptual standpoint, higher levels of academic buoyancy can moderate (i.e., buffer) the effects of academic challenges on subsequent outcomes [60, 76]. The observed model of moderating effects partially supports our third hypothesis. While higher academic buoyancy did not have a significant beneficial effect on test performance in the case of low EFL learning burnout, it mitigated the impact on test performance to some extent in the case of high burnout. That is, the higher the level of EFL learning burnout, the more that the value of higher academic buoyancy on academic performance was accentuated. This result implies that academic buoyancy acts as a safeguard against the negative impact of elevated burnout levels in foreign language learning on test performance. It also indicates that higher academic buoyancy does not protect test performance at lower levels of enjoyment but rather amplifies test performance at higher levels of enjoyment. This finding underscores that academic buoyancy helps reinforce the beneficial effects of EFL learning enjoyment, a positive emotion, on test performance. As Xu and Wang [63] mentioned that academic buoyancy fosters learning behaviors through the creation of a learning atmosphere where positive emotions are initiated.

Theoretically, academic buoyancy is associated with adaptive responses to adversity, including enhancing positive emotions and reducing negative emotions [59]. Furthermore, academic buoyancy reduces not only the intensity of emotions like anxiety and boredom, but also their adverse effects on academic achievement. The findings of this study showed that lower levels of academic buoyancy had little effect on the negative relationship between EFL learning burnout and test performance. As academic buoyancy increased, it buffered the negative effects of burnout, thus diminishing the negative relationship between burnout and test performance. That is, at lower levels of burnout, there was little difference in achievement between EFL students with lower academic buoyancy and those with higher academic buoyancy. However, as burnout increased, EFL students with higher academic buoyancy showed higher achievement than students with lower academic buoyancy.

Currently, there is a paucity of research related to learners’ academic buoyancy within the field of SLA. Limited exploration within educational psychology has explored the potential moderating role of academic buoyancy. Martin and Marsh [76] found a marginal impact of the interaction between academic buoyancy and academic adversity on subsequent academic adversity among secondary school students. Putwain et al. [59] identified that academic buoyancy moderates the relationship between anxiety and mathematics test performance among elementary school students. Test performance was highest when anxiety was low and academic buoyancy was high. Subsequently, Putwain et al. [77] found that academic buoyancy protects end-of-year test grades from minor adversities in upper secondary students. In our study, we extend this understanding, proposing that EFL academic buoyancy mitigates (or facilitates) the negative (or positive) effects of burnout (or enjoyment) on test performance. This is in line with the findings of Wu et al. [54], who highlighted the crucial role of resilience in mitigating the adverse effects of burnout among EFL learners. The following considerations may explain this pattern.

First, enjoyment is pivotal in EFL academic contexts, positively influencing students’ well-being, motivation, engagement, and psychological resilience [4, 78]. Academic buoyancy can be used to explain the above everyday academic resilience [57, 58]. As posited by Skinner et al. [79, 80], heightened academic resilience correlates with increased academic engagement, adaptive coping strategies, and enhanced academic persistence. Consequently, EFL students with higher academic buoyancy exhibit positive attitudes towards challenges, amplifying the favorable impact of enjoyment on test scores.

Second, the study found that high academic buoyancy also protects students’ test performance, especially as burnout increases and academic buoyancy provides more and more protection. This finding corroborates the buffering effect of academic buoyancy [76], in which the negative effects of negative emotions on academic performance are fully or partially mitigated in students with high academic buoyancy. Although less research has focused on the protective effect of academic buoyancy on academic performance in EFL, as Putwain et al. [81] found, the indirect negative relationship between test anxiety and test achievement is reduced in highly academically buoyant students. Further substantiating this protective effect, Hirvonen et al. [82] stated that high academic buoyancy is linked to reduced avoidance behaviors, fewer failure expectations, and higher task-oriented planning via academic emotions. High academic buoyancy was associated with low boredom and hopelessness, leading to lower expectations of failure. Thus, students with high academic buoyancy can cope with the ongoing challenges and frustrations presented to them [57, 58] and become more resilient in the process.

## Conclusion

This study constructed a moderating effect model of EFL learning emotions predicting test performance. We found that EFL learners’ learning enjoyment, boredom, burnout, academic buoyancy, and academic performance were significantly correlated. It concluded that academic buoyancy helped students cope with higher levels of EFL learning burnout. Academic buoyancy motivated students to perform better at higher levels of enjoyment. It also investigated the mechanism by which EFL learners’ emotions and academic buoyancy are linked to each other and jointly influence EFL academic performance. The possible moderating effect of academic buoyancy was further verified through an analysis of such an effect. This study reaffirms the good applicability of educational psychology and PP theories to EFL teaching research. The results suggest that to contribute to students’ academic achievement, EFL teachers need to actively pay attention to individual differences in students’ emotions and to their emotional changes; they also need to exert effort to create a positive EFL learning atmosphere for students while guiding them to face their negative emotions. Interventions such as structured emotional support programs and workshops aimed at building academic buoyancy among students can be particularly beneficial. Implementing professional development programs for teachers focusing on emotional intelligence and its application in the classroom can also equip instructors with the skills needed to manage and positively influence students’ emotional states.

Despite these implications, this study suffers its own limitations, too. First, due to the intricate nature of EFL learners’ emotions, it is advisable for future studies to utilize longitudinal studies to reveal the dynamic interplay between academic emotions and EFL proficiency [12], enhancing our nuanced understanding of this complex relationship. Second, the study relies on self-reported data, which may be subject to social desirability bias and affect the accuracy of the findings. Future research should incorporate multiple data sources, including observation and reflections, to validate the results. Third, the sample is limited to EFL learners in a specific cultural context, which may limit the generalizability of the findings to other EFL learning contexts. Further studies should consider diverse cultural settings to explore the universality of the observed patterns.

## Supporting information

S1 File (ZIP)
